# The dual role of CD6 as a therapeutic target in cancer and autoimmune disease

**DOI:** 10.3389/fmed.2022.1026521

**Published:** 2022-10-05

**Authors:** Mikel Gurrea-Rubio, David A. Fox

**Affiliations:** Division of Rheumatology, Department of Internal Medicine, University of Michigan, Ann Arbor, MI, United States

**Keywords:** CD6, CD318, ALCAM (CD166), NK cells, CD8 lymphocytes+, autoimmunity, cancer

## Abstract

Autoimmune disease involves loss of tolerance to self-antigen, while progression of cancer reflects insufficient recognition and response of the immune system to malignant cells. Patients with immune compromised conditions tend to be more susceptible to cancer development. On the other hand, cancer treatments, especially checkpoint inhibitor therapies, can induce severe autoimmune syndromes. There is recent evidence that autoimmunity and cancer share molecular targets and pathways that may be dysregulated in both types of diseases. Therefore, there has been an increased focus on understanding these biological pathways that link cancer and its treatment with the appearance of autoimmunity. In this review, we hope to consolidate our understanding of current and emerging molecular targets used to treat both cancer and autoimmunity, with a special focus on Cluster of Differentiation (CD) 6.

## Cancer and autoimmunity

Cancer cells can take advantage of physiologic immunoregulatory mechanisms to evade immune responses. Typically, a decrease in numbers and/or function of regulatory T and B cells, dendritic cells and M2 macrophages favors autoimmunity, while an increase in the same cell subsets or functions is associated with cancer progression. In most cancers, the immune infiltrate is composed of macrophages, T regulatory cells (Tregs), cytotoxic T cells and NK cells, whereas neutrophils and dendritic cells are typically found outside the tumor border (1). Autoimmune conditions are usually associated with increased T helper (Th) type 1 and 17 cells, and in some diseases infiltrates that also include B lymphocytes and plasma cells (2).

The tumor microenvironment (TME) is increasingly recognized as having a key role in tumor progression, largely due to the interactions between cancer, immune, vascular and stromal cells that take place in and around the tumor site. One distinct characteristic of the TME is its high levels of TGF-β (transforming growth factor beta), which is regarded as a key cytokine in promotion of immunosuppression (3). Tregs, which are typically characterized by an up-regulated expression of forkhead box P3 *(FoxP3)*, are found in high proportions within the TME of most cancers. These highly activated and immunosuppressive cells are known to infiltrate the lung, breast and pancreatic TME and have the ability to suppress cytotoxic CD8+ T cells, NK cells, NKT cells, M1 macrophages and the maturation of dendritic cells (4). The mechanisms by which Tregs suppress anti-tumor immunity remain largely undefined, although recent studies point out that most Tegs can suppress NK cells by (i) deprivation of IL-2, (ii) induction of apoptosis and (iii) generation of Indoleamine 2,3-dioxygenase (IDO) and adenosine (ADO) *via* the expression of CD39 and CD73 (5, 6). Tumor specific CD8+ T cell cytotoxicity is suppressed both *in vitro* and *in vivo* through TGF-β signals (7).

## Current therapeutic targets for autoimmunity and cancer

Autoimmunity is known to act as a substrate for the emergence of cancer and *vice versa*. Chronic inflammation causes DNA damage and creates an environment favorable for the development of cancer. Conversely, many patients with malignancies develop autoimmune and rheumatic manifestations, especially those who are treated with checkpoint inhibition therapies. Therefore, there has been a recent interest in developing new targets linked to molecular pathways and cell surface proteins that are known to be important in the development of both cancer and autoimmunity.

CD20 is expressed on the surface of mature B cells and is known to contribute to the activation and proliferation of B cells by intracellular tyrosine phosphorylation. Monoclonal antibodies against CD20 (rituximab and ofatumumab) have been successfully used for the treatment of B-cell lymphoma and some autoimmune diseases such as rheumatoid arthritis (8–11).

CXCR3 (GPR9/CD183) has also been studied as target for autoimmunity and cancer. This chemokine receptor is primarily expressed on CD4+ and CD8+ T cells, dendritic cells, NK cells and on non-immune cells such as fibroblastic, endothelial and epithelial cells (12). Among the CXCR3-binding chemokines, two groups can be distinguished based on their structural form. The first group is composed of CXCL9, CXCL10 and CXCL11, which are key immune chemoattractants during interferon-induced inflammatory responses. The second group is formed by the platelet-derived ligands CXCL4 and CXCL4L1, which are known to exhibit potent antiangiogenic activity and bind both CXCR3A and CXCR3B (12). The roles of CXCR3 ligands in autoimmunity have been extensively studied. In rheumatoid arthritis (RA), high CXCR3 expression on mast cells is associated with CXCL9 and CXCL10 expression in synovial fluid from RA patients but not in traumatic arthritis or osteoarthritis patients (13). Synovial CXCL10 expression is highly elevated in juvenile idiopathic arthritis (JIA) and, interestingly, raised levels of CXCL4 in plasma have also been observed in a subset of RA patients, specifically in those with vascular lesions.

The CXCR3 axis is also implicated in the pathogenesis of lupus nephritis and inflammatory bowel disease. For instance, in murine models of lupus nephritis, CXC3R directs pathogenic effector T cells into the kidney (14). In cancer, CXCR3 ligands, as well as several other chemokines, recruit innate and adaptive immune cells into the tumor microenvironment. For instance, CXCR3+ lymphocytes are attracted by CXCL9, which is secreted by stromal cells in gastric and ovarian cancer, thus facilitating the entrance of lymphocytes into the tumor sites. CXCL10 also potentiates the accumulation of CXCR3+ effector T cells, particularly CD8+ T cells, into the tumor microenvironment and directly suppresses tumor growth in various cancers (15, 16). Another important role of CXCR3 ligands in anti-tumor immunity relies on the effectiveness of CXCL4 and CXCL4L1 (factors produced by platelets) at inhibiting lymphangiogenesis and preventing metastatic escape of tumor cells *via* the lymphatic vasculature (17).

The pro-inflammatory cytokine interleukin-6 (IL-6) is another example of a therapeutic target that has been linked to both autoimmunity and cancer. The IL-6 signaling cascade is known to play a role in Th17 differentiation (18). Several IL-6 blocking antibodies have been approved for the treatment of rheumatoid arthritis. More recently, because the IL-6-STAT3 signaling pathway can be directly involved in progression and metastasis of prostate cancer, evaluation of IL-6 and its receptor as targets for cancer treatment is currently being explored (19).

Celecoxib and rofecoxib are two cyclooxygenase-2 (COX-2) inhibitors that have been used to relieve the inflammation and pain of patients with osteoarthritis (OA) and rheumatoid arthritis (RA). These inhibitors and are also found to extend the overall survival of stage III colon cancer patients and non-small cell lung cancer patients (20). There is, however, a concern regarding the use of COX-2 inhibitors and the risk of developing cardiovascular disease.

The recent success of immune checkpoint inhibitors for the treatment of cancer has encouraged researchers to assess the potential use of checkpoint agonists for the treatment of autoimmunity. Currently, there are multiple clinical trials investigating these therapies. Strategies to treat autoimmunity safely and successfully through manipulation of checkpoint interactions will need to consider the multiple ligands and receptors pertinent to these targets.

## Novel therapeutic targets for autoimmunity and cancer: CD6

CD6 is a 105 to 130 kDa type I transmembrane glycoprotein belonging to the highly conserved scavenger receptor cysteine-rich superfamily (SRCR-SF). It is expressed by virtually all T cells, a subset of NK cells, B-lymphocyte B1a subsets, immature B cells and certain regions of the brain. Structurally, CD6 has an extracellular region composed of three SRCR domains and an intracellular portion with sites for the phosphorylation and recruitment of signal transduction proteins (21). Functionally, CD6 is involved in lymphocyte activation and differentiation upon adhesive contacts with antigen-presenting cells (APCs). Thus, far, CD6 has two known ligands: CD166 (activated leukocyte cell adhesion molecule or ALCAM) and CD318 (CDCP1, TRASK, SIMA135, or gp140). ALCAM/CD166 is expressed on activated T cells, monocytes, endothelium, epithelial cells and synovial fibroblasts. CD318 is expressed on epithelial and tumor cells with which T cells interact, but not by immune cells (22).

## CD6-ALCAM interaction

The first known ligand for CD6 was the Activated Cell Adhesion Molecule (ALCAM), also known as CD166. ALCAM is a cell surface glycoprotein related to the immunoglobulin superfamily molecules. It can be proteolytically cleaved by metalloproteases, causing its ectodomain to shed (23). The interaction between CD6 and its ALCAM (CD166) is important during immune development and in alloreactivity. It stabilizes the adhesive contacts established between T cells and APCs and optimizes subsequent proliferative and differentiation responses. In recent years, the CD6-ALCAM axis has been implicated in the pathogenesis of multiple autoimmune diseases and cancer (24).

CD6 was first identified and validated as risk gene for multiple sclerosis (MS) in 2009 and, soon after, the CD6-ALCAM axis was shown to play a crucial role in the pathogenesis of this disease. In a large genetic study of CD6, ALCAM and neuroinflammation, a polymorphism of ALCAM (rs579565G>A) and two SNPs of CD6 (rs17824933C>G and rs12360861G>A) were found to be strongly associated with risk, development and progression of MS. The risk of MS for AA individuals in rs12360861 was three-fold lower in comparison to GG individuals. Furthermore, significantly lower expression of CD6 mRNA was found in lymphocytes from MS patients compared to healthy individuals (25).

Knockout mice of CD6 and its ligands have been extensively studied in models of inflammation and autoimmunity. For instance, *CD6*^−/−^ mice are protected from experimental autoimmune encephalomyelitis (EAE), an animal model of autoimmune inflammatory diseases of the central nervous system commonly used for the study of MS. In these mice, CD6 deficiency leads to reduced Th1 and Th17 polarization and provides protection from spinal cord demyelination *in vivo* (26). *ALCAM*^−/−^ mice, however, develop a more severe autoimmune encephalomyelitis, due to an increased permeability of the brain blood barrier. This is believed to be caused by a dysregulation of junctional adhesion molecules with which ALCAM indirectly binds, suggesting an important function of ALCAM in maintaining the blood brain barrier integrity (27).

The CD6-ALCAM pathway is also implicated in the pathogenesis of lupus nephritis (LN). In mouse models of lupus, blockade of CD6 with an anti-mouse CD6 monoclonal antibody decreases T cell infiltration, cytokine levels and overall renal pathology (28). Furthermore, a recent study has shown that ALCAM and CD6 are elevated in patients with LN. High numbers of CD6+ cells were found exclusively in T cells from patients with LN, while ALCAM was expressed by renal cells, also at higher levels compared to normal individuals. Notably, itolizumab is currently being evaluated for safety, pharmacokinetics and pharmacodynamics for the treatment of LN. Itolizumab is an IgG1 monoclonal antibody that targets the extracellular SRCR distal domain 1 of CD6. Itolizumab has been shown to significantly reduce differentiation of T cells to Th17 cells and decrease production of IL-17 in patients with psoriasis in India (24, 29).

Similar to MS and LN, rheumatoid arthritis (RA) is another autoimmune disease in which CD6 is involved in its pathogenesis. CD6 and its ligands are strongly expressed in RA joints and are important for the initiation and maintenance of collagen induced arthritis (CIA), a T cell driven mouse model for RA. *CD6*^−/−^ mice develop lower clinical arthritis scores than wildtype (WT) mice, proving that CD6 can tune the severity of joint inflammation in these mice. Collagen-specific Th9 and Th17 are also decreased in *CD6*^−/−^ mice, as well as multiple pro-inflammatory joint cytokines. Unsurprisingly, treatment with UMCD6 (anti-CD6) in CD6 humanized mice, displays similarly effective responses in reducing joint inflammation in CIA (30).

Sjogren's syndrome and inflammatory bowel disease are two other autoimmune diseases in which CD6 and its ligand ALCAM (CD166) might play an important role in their pathogenesis. In Sjögren's syndrome, ALCAM is overexpressed in the salivary glands of these patients and the use of itolizumab as therapeutic option has been proposed for clinical trials (31). Similarly, the expression of both CD6 and ALCAM (CD166) is markedly increased in the inflamed mucosa of inflammatory bowel disease patients compared with normal controls (32–34).

In cancer, ALCAM has been linked to poorer prognosis and higher metastatic risk in some cancers such as breast, thyroid, head and neck and liver cancer (35). ALCAM-positive cells have superior sphere-forming ability and cancer-initiating potential in prostate cancer and promote evasion of apoptosis in breast cancer. Specifically in breast cancer, high expression of ALCAM occurs in approximately 50% of patients with triple-negative cancers and around 80% in patients with estrogen receptor positive, human epidermal growth factor receptor 2 (HER2) tumors (36). Praluzatamab ravtansine or CX-2009, an activated antibody-drug conjugate that targets ALCAM, is currently undergoing phase II clinical trials for the treatment of breast cancer (36). Notably, a single-chain antibody named scFv173, which recognizes ALCAM, has been shown to reduce ALCAM-mediated adhesion in breast cancer, both *in vitro* and *in vivo* (35). ALCAM is also up-regulated in liver tissue and serum from patients with hepatocellular carcinoma (HCC). In pancreatic cancer, patients whose circulating cancer cells have high levels of ALCAM, tend to have significantly shorter survival. Moreover, high levels of ALCAM in colorectal tumors are strictly associated with poorer survival, nodal status, tumor grade and high risk of metastasis (29). Use of ALCAM targeted therapeutics for cancer might raise issues of off target consequences related to widespread expression of ALCAM on other cell types including many types of cells of the immune system.

However, ALCAM expression is not always linked to bad prognosis in cancer. In sarcomas, the vast majority of patients with high levels of ALCAM have more favorable outcomes and tend to remain metastasis free (37). Similarly, in a recent multivariate survival analysis in patients with non-small cell lung cancer, expression of ALCAM strongly correlated with a better prognosis/longer patient survival (38).

## CD6-CD318 interaction

Cub domain-containing protein 1 (CDCP1), also known as CD318, is a transmembrane glycoprotein expressed by fibroblasts and the epithelium of normal and cancer cells. CD318 is phosphorylated by Src family kinases before recruiting and activating protein kinase C delta type (PKCδ) (39).

In rheumatoid arthritis (RA), both known CD6 ligands, ALCAM and CD318, are known to participate in adhesion of T cells to fibroblast-like synoviocytes (FLS) derived from RA synovial tissue. Moreover, soluble CD318 (sCD318) is found in RA synovial fluid at levels higher than in normal or RA serum, and sCD318 is chemotactic for T cells at concentrations similar to the levels found in RA synovial fluid (22). As in *CD6*^−/−^ mice, *CD318*^−/−^ mice are also protected from autoimmune encephalomyelitis and collagen induced arthritis. In both *CD6*^−/−^ mice and CD6-humanized mice treated with UMCD6 (mouse anti-human CD6 mAb), striking reductions in clinical signs of disease, pathogenic Th1/Th17 responses, and inflammatory cell infiltration into the target organs are observed, making the CD6-CD318 axis a novel and exciting regulator of T-cell driven autoimmunity (22). Similar studies have been done to examine the role of CD6 and its ligand CD318/CDCP1 in autoimmune uveitis (EAU). In these studies, *CD6*^−/−^ mice with EAU had significantly decreased retinal inflammation and reduced autoreactive T cell responses, indicating that CD6-targeted therapies are a promising new treatment for uveitis (33, 34).

The role of CD318 (CDCP1) in cancer has been extensively explored. Overexpression of CD318 (CDCP1) uniformly correlates with poor overall survival in lung, colon, ovarian, breast, renal, hepatocellular, acute myeloid leukemia and pancreatic cancers, partly due to its involvement in tumor metastasis formation *via* Src. The first hints suggesting a link between CD318 and Src family kinases in cancer metastasis, came from studies of pharmacological inhibition of Src with PP2 and dasatinib. In these studies, both Src inhibitors strongly suppressed the growth of CD318-overexpressing cells in 3D cultures of soft agar (40).

Not only does CD318 (CDCP1) play an important role in the initiation of metastasis, but it also mediates cancer progression by altering cancer cell growth. Multiple studies have now demonstrated that CD318 expression is involved in cancer cell growth through its interaction with receptor tyrosine kinases (RTKs) and HER2 signaling pathways and their subsequent downstream proteins Ras, Src and AKT. For instance, the interaction between CD318 and HER2 promotes *in vitro* colony formation and *in vivo* orthotopic tumor growth in several models of breast cancer (41). In non-small lung cancer, Ras induces the expression of CD318 and drives Src-mediated survival in *in vitro* models of lung cancer (42). In ovarian cancers, CD318 mediates spheroid growth and is involved in the activation of AKT (43). Interestingly, some studies in pancreatic cancer also support the role of CD318 in cell migration and matrix degradation *via* different molecular mechanisms involving Src, PKCδ and MMP9 (27, 44).

Soon after CD318 was recognized as a second ligand of CD6, efforts were made to understand its potential role in anti-tumor immunity. Recent studies on the potential involvement of CD318 in anti-tumor immunity indicate that disrupting CD6-CD318 interaction is highly effective in experimental systems as a novel cancer immunotherapy. In co-culture experiments with cell lines derived from human triple-negative breast cancer, non-small-cell lung cancer, and prostate cancer, substantial enhancement of cancer cell death and reduced survival of cancer cells was found in the presence of human lymphocytes and UMCD6 (anti-CD6) (38). Importantly, this effect was consistently more robust than the effect of either pembrolizumab or nivolumab, two monoclonal antibodies against the Programmed Cell Dead Protein 1 (PD-1). The augmentation of lymphocyte cytotoxicity by targeting the CD6-CD318 axis with UMCD6 is due to direct effects of anti-CD6 on NK cells and CD8+ T cells when CD6 is internalized. Both *in vitro* and *in vivo*, multiple gene expression changes are seen in CD8+ T cells and NK cells treated with UMCD6, along with changes in the levels of the corresponding proteins, all of which are consistent with enhanced cytotoxic capabilities such as over-production of granzyme-b, perforin and activating receptors (e.g., NKG2D) and down-regulation of inhibitory receptors (e.g., NKG2A). Moreover, UMCD6 exerted similar effects *in vivo* in a human breast cancer xenograft system in immunodeficient mice. In those experiments, mice that received UMCD6 showed marked shrinkage of tumors by 1 week, accompanied by an aggressive infiltration of lymphocytes, dominated by NK cells (38). Using a different approach, high doses of recombinant CD6 that masked CD6 ligands *in vivo* generated a modest anti-cancer effect (45).

Despite CD318 being expressed in normal tissues, several findings suggest that toxicities associated with inhibition of its interactions with CD6 in patients with cancer, will be clinically manageable. CD318 knockout mice develop and reproduce normally and are protected from T cell driven autoimmunity, indicating that its functions are not essential for normal physiology. In contrast, humans or mice that lack PD-1 or cytotoxic T lymphocyte-associated antigen 4 (CTLA-4) exhibit a global autoimmune diathesis that corresponds to the toxicities observed when these structures are targeted in immunotherapy of cancer. A monoclonal antibody against CD318 has also been studied in the context of anti-tumor autoimmunity. Anti-CD318 produces a more modest effect on cancer cell death and survival compared to UMCD6 (anti-CD6). The more modest effect of anti-CD318 could be attributable to a dual effect of UMCD6: rapid internalization of CD6 prevents or reverses engagement of CD6 by its ligands on cancer cells, and UMCD6 also directly activates the cytotoxic properties of CD8+ and NK cells, whereas anti-CD318 does not have any effect on lymphocyte cytotoxicity (36, 52).

**Figure 1:**
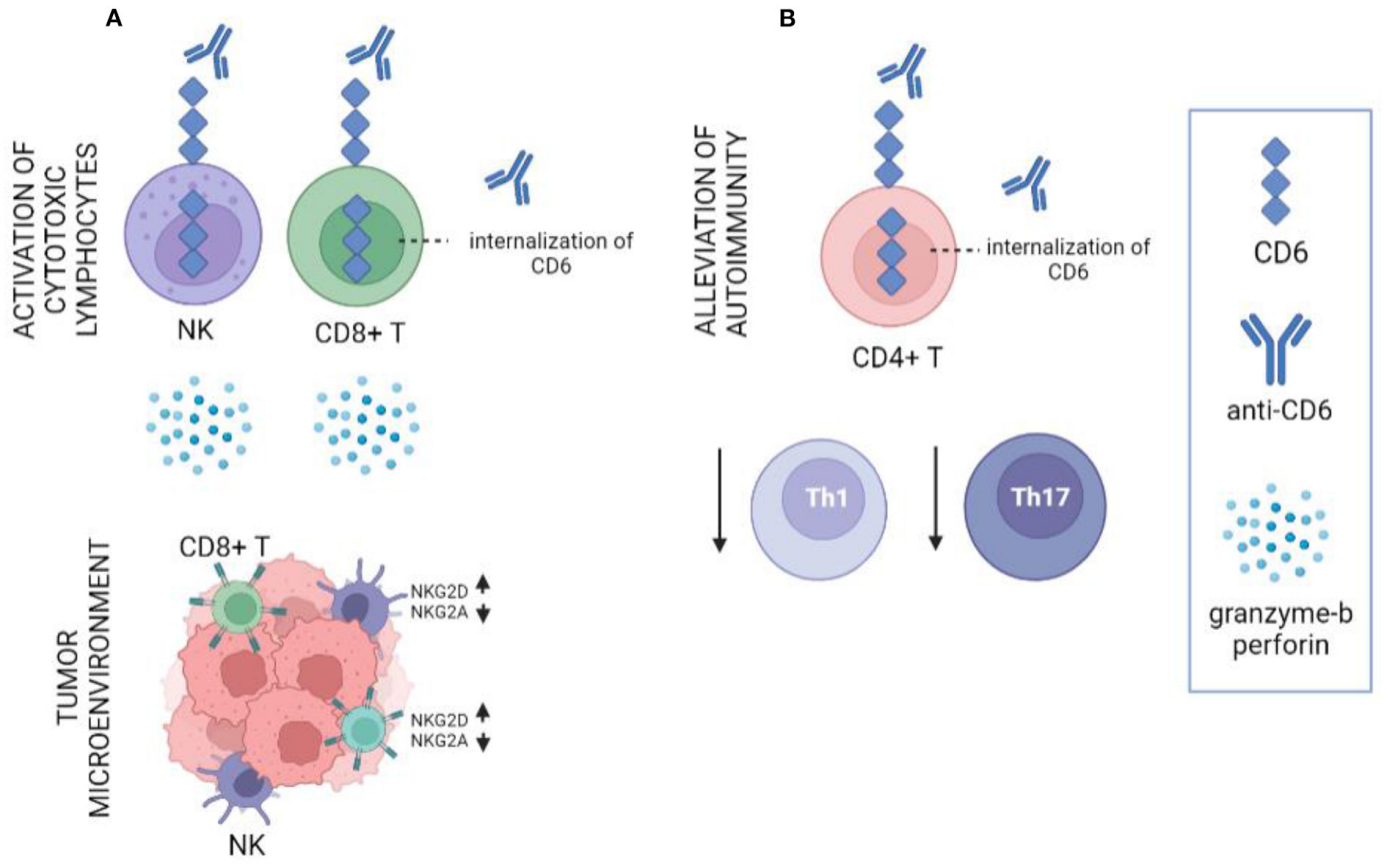
Schematic representation of the role of anti-CD6 (UMCD6) in cancer and autoimmunity. **(A)** Internalization of CD6 on CD8+ T cells and NK cells with UMCD6 induces the activation of these cells by up-regulation of granzyme-b, perforin and the activating receptors NKG2D, and down-regulation of the inhibitory receptor NKG2A. **(B)** UMCD6 provides a protective effect in autoimmune diseases by suppression of differentiation of effect Th1 and Th17 cells.

These findings point out that targeting CD6 and its ligand CD318 could both suppress autoimmune diseases through its effects on differentiation of effector CD4 cell subsets, while also activating the anti-cancer cytotoxic properties of CD8+ and NK cells, creating the potential for an approach to cancer immunotherapy that would suppress rather than instigate serious autoimmune diseases (Figure 1).

## Conclusion

The association between autoimmunity and cancer is an area of particular interest for oncologists and rheumatologists, especially after the introduction of checkpoint inhibitors for the treatment of cancer. Many autoimmune disorders and immunosuppressive therapies have been linked to an increased risk for cancer. And many cancer patients develop autoimmune disorders as a result of cancer treatments. Currently, there are only a few approved therapeutics capable of treating both autoimmunity and cancer. These therapies presented above may have additional applications in both autoimmunity and cancer. With other molecular targets, such as CTLA-4, the approaches to treatment of cancer vs. autoimmunity are opposite, agonistic vs. antagonistic. Pre-clinical studies, involving the CD6-CD318 axis, and an anti-CD6 monoclonal antibody, suggest new strategies that could treat autoimmunity and cancer simultaneously, through distinct effects on subsets of lymphocytes CD6 blockade concurrently reduces Th1 and Th17 differentiation and increases T cell and NK cell cytotoxicity. Better understanding of new mechanisms connecting autoimmunity and cancer provide hope that these two conditions can be successfully treated simultaneously.

## Author contributions

All authors listed have made a substantial, direct, and intellectual contribution to the work and approved it for publication.

## Conflict of interest

The authors declare that the research was conducted in the absence of any commercial or financial relationships that could be construed as a potential conflict of interest.

## Publisher's note

All claims expressed in this article are solely those of the authors and do not necessarily represent those of their affiliated organizations, or those of the publisher, the editors and the reviewers. Any product that may be evaluated in this article, or claim that may be made by its manufacturer, is not guaranteed or endorsed by the publisher.
